# *Flow Plex*—A tool for unbiased comprehensive flow cytometry data analysis

**DOI:** 10.1002/iid3.246

**Published:** 2019-04-23

**Authors:** Johannes Nowatzky, Ezra Resnick, Julia Manasson, Cristy Stagnar, Arshed Fahad Al‐Obeidi, Olivier Manches

**Affiliations:** ^1^ Department of Medicine, Division of Rheumatology NYU School of Medicine New York New York; ^2^ Google, Inc New York New York; ^3^ Recherche et Développement, Immunobiology and Immunotherapy in Chronic Diseases Institute for Advanced Biosciences, Inserm U 1209, CNRS UMR 5309, Université Grenoble Alpes, Etablissement Français du Sang Auvergne‐Rhône‐Alpes Grenoble France

**Keywords:** computation, data analysis, flow cytometry

## Abstract

**Introduction:**

The information content of multiparametric flow cytometry experiments is routinely underexploited given the paucity of adequate tools for unbiased comprehensive data analysis that can be applied successfully and independently by immunologists without computational training.

**Methods:**

We aimed to develop a tool that allows straightforward access to the entire information content of any given flow cytometry panel for immunologists without special computational expertise. We used a data analysis approach which accounts for all mathematically possible combinations of markers in a given panel, coded the algorithm and applied the method to mined and self‐generated data sets.

**Results:**

We developed *Flow Plex*, a straightforward computational tool that allows unrestricted access to the information content of a given flow cytometry panel, enables classification of human samples according to distinct immune phenotypes, such as different forms of autoimmune uveitis, acute myeloid leukemia vs “healthy”, “old” vs “young”, and facilitates the identification of cell populations with potential biologic relevance to states of disease and health.

**Conclusions:**

We provide a tool that allows immunologists and other flow cytometry users with limited bioinformatics skills to extract comprehensive, unbiased information from flow cytometry data sets.

## 1 INTRODUCTION

Flow cytometry is a powerful tool enabling high‐throughput, multiparametric assessment of biological properties at the single cell level. However, conventional data analysis approaches have relied mostly on the retrieval of data from bivariate plots each combining two markers of interest to the question investigated, and subsequent gating on further subpopulations defined by additional markers. This allows targeted assessment of predefined cell populations, which in most cases are known to the investigator, but leaves the overwhelming majority of information obtainable from a given panel of markers unaccounted for. In fact, the information content that can be retrieved from any given panel increases exponentially with the number of markers used in the panel as each maker can assume at least two mutually exclusive conditions: positive or negative. Considering that each given marker in the panel may or may not be taken into account to depict a cell population, the number of cell populations that can be described by the panel equals ncp = 3^nm^–1, where ncp = number of cell populations and nm = number of markers (‐1 theoretical possibility when no marker is chosen). For example, this accounts for 80 informative populations for four markers, 728 for six markers, and 59 048 for 10 markers, thus providing a considerable amount of information. In contrast, conventional approaches focus on the identification and measurement of a few populations defined by the sequential combination of markers.^1^ Computed clustering approaches of cell populations, spearheaded by the FlowCAP^2^ (Flow Cytometry: Critical Assessment of Population identification methods) initiative, have ameliorated the efficiency in flow cytometry data analysis.^3^ Computational methods such as the ones implemented by flowType/RchyOptimyx,^4^ FlowCAP,^5^ Citrus,^6^ SWIFT,^7^, ^8^ or COMPASS,^9^ further aim at identifying cell types or biomarkers important to predict survival or differentiate clinical samples, starting from high dimensional flow cytometry data. These approaches have shown considerable success in classifying and predicting disease states and clinical evolution, as evidenced by the FlowCAP II and IV challenges.^2^ However, these methods require intermediate to advanced bioinformatics skills (such as in R, Matlab, or Python), which still represents a hurdle for their widespread use for research or clinical purposes. Web‐based platforms provide only partial alternatives for nonprogrammers, such as GenePattern (Flow Cytometry Suite) allowing cell population clustering analysis, or the commercial interface Cytobank that helps with feature extraction and data structuring. Our aim here is to provide the routine flow cytometry user with a simple, directly applicable tool to extract all available information derived from positive/negative (i.e., ideally fluorescence minus one [FMO]‐based) gating of cell populations, without computational expertise. We show that this methodology allows efficient partitioning of clinical samples using only a few widely used markers.

## 2 METHODS

The supplement section of this paper contains all the information necessary for the independent reproduction and application of this tool, including the source code of the program.

### 2.1 Acquisition and processing of human samples

Leukocytes were purchased from the New York Blood Center (Component Laboratory, Long Island City, NY) which routinely obtains informed consent from all subjects and approved all experimental protocols. All methods were carried out in accordance with relevant guidelines and regulations.

### 2.2 Sample processing and flow cytometry

Peripheral blood mononuclear cells were obtained through centrifugation over Ficoll (GE Healthcare, Uppsala, Sweden) and cryopreserved on the day they were received. Cells from all recruited donors were thawed, stained with the antibodies specified in Table S1 for 20 minutes at room temeparture, fixed in 4% paraformaldehyde and acquired on an LSRII the following day. The staining panel for this experiment was optimized in various single‐color titration and add‐in experiments as described.^10^ N‐1 (FMO) controls were obtained to guide gating decisions. Flow cytometry analysis was performed using FlowJo software.

### 2.3 Human study subject recruitment and sample selection for analysis

We aimed to collect samples from female and male humans in two age groups: 8 to 35 years old, and 65 to 95 years old and requested leukocyte enriched blood from presumably healthy human donors with these age and sex specifications from a commercial provider. We received 32 samples (equally split between male and female donors) in response to this request over a period of 7 months. The commercial provider of the blood samples allows verification of subject‐related health data retrospectively. All samples were verified to have the requested age and sex specifications, except two samples which were outside the requested age range (HDC‐39 and HDC‐40). For one sample, the precise age could not be reliably verified (HDC‐38). These samples were excluded. As classification was not satisfactory when middle‐aged donors were included in the analysis, we decided to exclude all donors above the age of 30 to escalate the age difference (HCD‐26, ‐27, ‐28, ‐33, ‐39, ‐40, ‐49, and ‐52). Included study subjects are enlisted in Table S2. Serological test results for all donors were provided as well. All donors tested negative for HBC, HCV, HTLV, Chagas disease, and Zika virus. Forty‐seven percent of the included donors were seropositive for CMV, consistent with the expected CMV seropositivity rate in presumably healthy human subjects in the general population.

### 2.4 Computation of subpopulations

The algorithm calculates all subpopulations for *M* markers using coding elements (the lowest level gated populations) and intermediate gated populations as input.

The number of subpopulations can be calculated as follows for four markers as an example:

Each marker defines one positive and one negative population (2^1^), and in general 2*^m^* populations are defined for *m* markers. Enumerating the subpopulations involves going through all populations defined by one marker, two markers, and so forth. The number of marker combinations when choosing m markers among 4 is *C*(4,*m*) = 4!/(*m*!(4−*m*)!), with markers defining 2*^m^* populations.

Thus, the total number of populations is *C*(4,1) 2^1^ + *C*(4,2) 2^2^ + *C*(4,3) 2^3^ + *C*(4,4) 2^4^ = 80, and in general *C*(*M*,1) 2^1^ + *C*(*M*,2) 2^2^ + … + *C*(*M*,*M*) 2*^M^* = 3*^M^*−1 populations for *M* markers.

**Figure 1:**
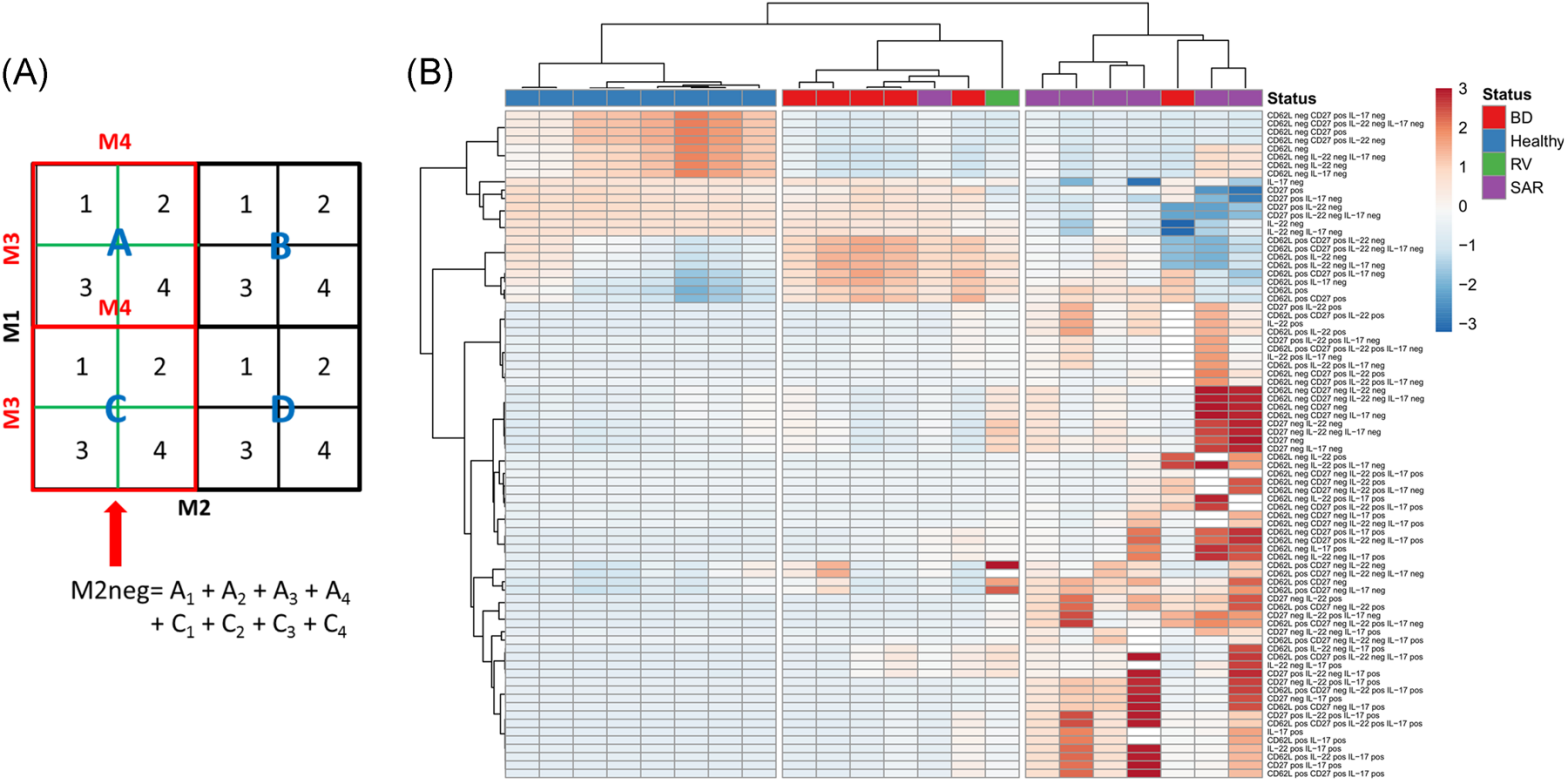
Exhaustive enumeration of immune subpopulations by flow cytometry for disease classification. A, In a four‐marker (M1‐M4) flow cytometry panel, subpopulations are defined by negative/positive thresholds (quadrants). Coding elements are derived by nested gating of M3, M4‐defined subpopulations into M1, M2‐defined populations. The algorithm combines these coding elements to calculate the representation of all possible subpopulations, exemplified here for the M2‐negative population. B, Hierarchical clustering of healthy (blue), Behçet's disease (BD‐red), sarcoidosis (SAR ‐ purple) or Retinal Vasculitis (RV‐green) according to all subpopulations of immune cells in peripheral blood mononuclear cells defined by CD62L or CD27 expression, and IL‐22, IL‐17A secretion. Values in the color legend represent *z*‐scores after autoscaling subpopulation percentages. IL, interleukin

Populations are computed from coding elements and higher level gated populations. Thus for four markers, the population *A*2/4 (*M*1+, *M*2−, *M*4+) (Figure 1) is computed as %(*A*2 + *A*4) × %(*A*)/100, where %() is the percentage of the parent population.

### 2.5 Workflow

For data entry, nested gating is performed in FlowJo (FlowJo LLC, Ashland, OR), and populations are entered into the “Create Table” module from higher to lower gating levels (Figure S7) (see the Supporting Information file “instructions for running the program” for more details).

A table is created for all samples, gathering population values in the specified order. The resulting table is pasted into the Java‐created.csv input file (named “4markers.csv” in the section describing “Running the program”) (Figure S8, top).

From the input file, the Java program generates an output.csv file containing a table of all population values for each sample (named “4markers_output.csv” in the section describing “Running the program”) (Figure S8, bottom).

To demonstrate the impracticality of performing calculations for even small numbers of markers, we computed output values under the application of the above‐mentioned principles for four markers in excel spreadsheets. An example is attached in (Figure S9) that exemplifies the summation of coding elements into final output values.

### 2.6 Programming

The Java program recursively generates all 3*^n^* present/absent/undefined combinations of the markers, then computes the percentage value for each combination by recursively summing the present/absent subtrees for every marker that is undefined. The value for a combination where all markers are defined is computed directly based on partial sums of the input percentage values.

Instructions to run the program are attached as a Supporting Information file “Instructions for Running the Program”. The source code is provided in two Supporting Information files MarkerCombosInputGeneratorFlowJo.java and MarkerCombosBatch.java. Two versions are included, depending on whether the input tables are comma‐separated files (decimal point format, e.g., in the United States) or semicolon separated files (decimal comma format, e.g., in most of Europe).

### 2.7 Verification of computations

To verify the accuracy of computed population values, they were directly compared with manually gated subpopulation statistics. As shown in Figures S1 and S2, there was concordance between calculated values and direct gating, with both the formula‐based spreadsheet (Figure S1) and the Java program (Figures S1 and S2).

### 2.8 Principle component analysis (PCA) and hierarchical clustering

For principle component analysis and hierarchical clustering, data were entered into the ClustVis online program (http://biit.cs.ut.ee/clustvis/).^11^ Pearson correlation was used as the distance measure.

For some supplementary analyses, hierarchical clustering was performed based on Euclidian distance applying default specifications of the CIMMiner online tool at (https://discover.nci.nih.gov/cimminer/oneMatrix.do).

PCA and hierarchical clustering analyses were replicated in R using prcomp and heatmap.2 from the gplots packages (not shown).

### 2.9 Ethical approval

All necessary ethical approval for data presented in this study has been acquired.

## 3 RESULTS

We implemented an approach to compute data sets generated through a minimal number of bivariate gating steps to generate metadata describing accurately the entire set of cell populations that can be retrieved from a panel, when each marker used in the panel is defined as either positive or negative, thus extracting the full information content obtainable from each gating choice. This approach was pioneered and applied in a low throughput setting by Hofmann et al,^12^ and later incorporated into the flowType algorithm available on Bioconductor for R users by Aghaeepour et al. The program we have developed computes values representing the frequencies of all possible subpopulations when all markers in the panel are taken into consideration through multiplication of the frequencies of all back‐gated subpopulations toward the starting population (Figure 1A).

We called the numeric values for these populations “coding elements” as further computation was based on their rearrangement and combination. These coding elements depict the highest level of resolution for the differential representation of cell populations in the panel. Extraction of coding elements can be conveniently done and exported using the popular flow cytometry analysis software FlowJo, or can be performed on any other analysis platform. In the next step, coding elements are sequentially paired to determine the representation of each mathematically possible cell population within the starting population when all possible *combinations* of markers *and* their respective *bi‐modal qualities* (positive/negative) are taken into account. This involves the summation of coding elements specific to the representation level of the markers in the hierarchy of the gating tree. It is to be noted that this calculation cannot be performed easily by the current version of FlowJo or any similar flow cytometry analysis software. To enable the combinatorial population generation process for analysis of larger panels, we coded the algorithm in Java. For easy reference, we called the program *Flow Plex*.

To verify the accuracy of the obtained meta‐data sets using this approach, we generated and mined flow cytometry data. Percentage values for computable cell populations were obtained through manual gating. There was an excellent correlation between computed and manually gated populations (Figures S1 and S2).

Next, we asked whether this approach could help classify biological samples according to immune phenotypes. To this end, we first mined a data set generated from the peripheral blood of healthy humans and subjects with a uniform ocular pathophenotype (pan‐uveitis) caused by two different autoimmune diseases: Behçet's disease (BD) and sarcoidosis (SAR).^13^ Currently, no peripheral blood‐based diagnostic test can reliably discriminate between these disorders. Peripheral blood mononuclear cells gated for CD3^+^ CD4^+^ cells had been stained for CD62L, CD27, and for secretion of interleukin‐22 (IL‐22) and IL‐17A. Nested gating allowed measurements of all 80 coding elements derived from these four markers (Figure S3). Using the freely available online tool ClustVis,^11^ hierarchical clustering of samples based on these populations led to an accurate separation between healthy and diseased samples (Figure 1B). Furthermore, BD and SAR samples were classified with only minimal error, despite these disorders displaying a uniform ocular pathophenotype, that is, uveitis. Retinal vasculitis—a phenotype which is part of the clinical spectrum of both disorders but more characteristic of BD clustered with the latter (Figures 1 and S4). No single marker or subpopulation by itself could unambiguously differentiate between samples, thus supporting the use of multipopulation analysis for efficient classification.

**Figure 2:**
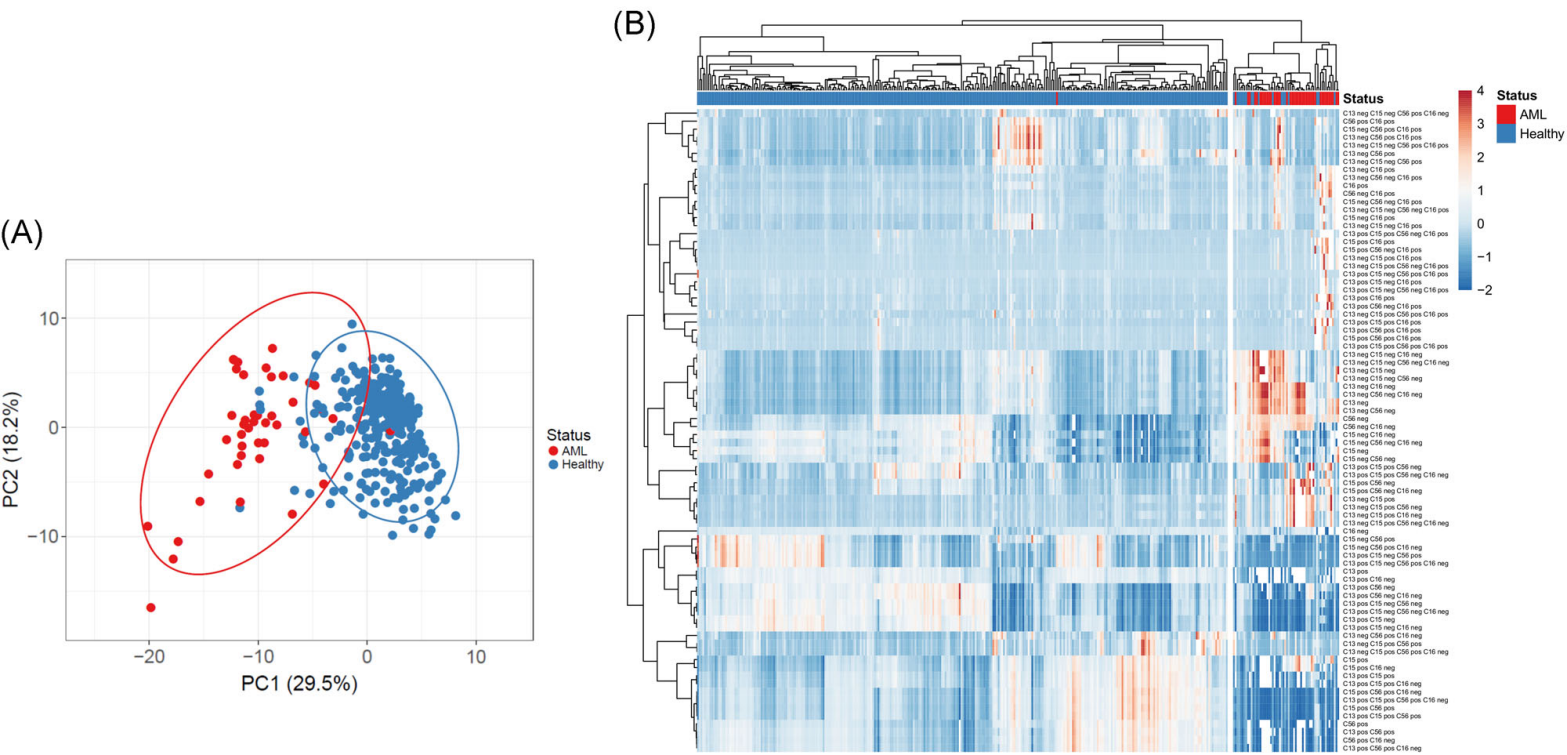
Separation of healthy and acute myeloid leukemia (AML) samples. A, Principal component analysis of healthy and AML samples characterized by all combinations of CD13, CD15, CD16, and CD56 expression. B, Hierarchical clustering of healthy and AML patient samples based the representation of cell populations defined by all possible marker combinations

We also tested the approach on another mined data set,^6^ to differentiate acute myeloid leukemia (AML) patients' samples (n = 43) from healthy donors' samples (n = 317). Several sets of markers were available to characterize identical samples. We focused on the combination of four markers: CD13, CD15, CD16, and CD56 (Figure S5), from which the algorithm generated 80 subpopulations. AML samples and healthy donor samples segregated in different regions by principle component analysis (Figure 2A), with only one AML outlier inside the healthy sample region. Hierarchical clustering using the 80 coding elements defined by the four markers clusters separated all but one AML sample from the healthy samples. This combination of markers allowed a small fraction (5.6%) of healthy samples to segregate with AML samples.

**Figure 3:**
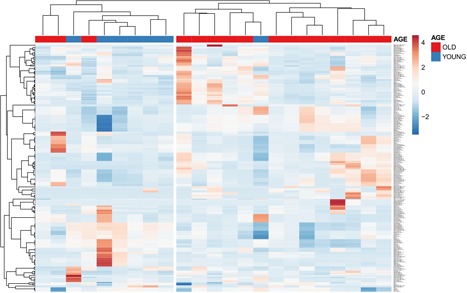
Hierarchical clustering of elderly (>65 years; red) and young (<30 years; blue) presumably healthy donor‐derived leukocyte samples according to 728 computed subpopulations in peripheral blood mononuclear cells defined by CD3, CD4, CD8, CD45RA, CCR7, and CD38 expression gated on CD127^lo^/CD25^hi^ lymphocytes

Finally, we applied the approach to one of our own data sets generated from presumably healthy donors of both sexes and different age groups (18‐30, n = 7, and 65‐95 years, n = 15). We created a panel incorporating common, well‐known immune‐markers: CD3, CD4, CD8, CD45RA, CCR7, and CD38 and gated on a CD127^lo^ CD25^hi^ lymphocyte starting population which contains the bulk of human regulatory T cells (an example of a six‐marker gating and nesting strategy is shown in Figure S6). The input of gated coding elements into the algorithm yielded the expected 728 populations. Despite the relatively low number of samples and the expected subtle differences in immune parameters between young and elderly populations, hierarchical clustering allowed very good categorical separation of samples (Figure 3). Again, no single marker was able to uniquely separate samples in an unambiguous way.

## 4 CONCLUSIONS

The approach described herein allows for the full exploitation of information available from a flow cytometry panel with minimal gating effort and is facilitated by *Flow Plex,* a computational tool we have developed for this purpose. For instance, for an eight‐marker panel, only four gating decisions need to be made and batched: one at each level involving two markers in the gating hierarchy—yielding a total of 3^8^−1 = 6560 different cell populations. The information obtained through this approach increases exponentially with the number of markers in the panel while the number of necessary gating decisions increases only linearly. This results in a vast increase in information over conventional gating. The latter—with increasing numbers of markers used—yields a constantly decreasing fraction of the information that can actually be obtained from the same panel. *Flow Plex* can function as a pipeline for the generation of high dimensional data by immunologists familiar with standard flow cytometry analysis software such as FlowJo, but without specialized computational training. These high‐volume data sets can then be analyzed employing straightforward routine approaches, such as conventional principal component analysis and hierarchical clustering techniques to group peripheral blood samples by states of health, disease or other variables/categories of interest, identify outliers and point to populations of potential interest. These tools are easily accessible online (e.g., the Clustvis website utilized here) and/or part of the standard repertoire of statistical support typically available to immunologists. Clustering of populations can help with the biological interpretation, which may be of interest for further discriminative and mechanistic investigations. Additional methods can be used to test the statistical significance of population proportion differences if desired. As an example, the uncertainty/robustness of clustering for the AML/ healthy data set (used as a training set for the program in the workflow) was measured by bootstrapping using the R package pvclust^14^ (Figure S10). Investigation of cell populations with importance in disease can be performed through statistical testing with correction for multiple comparisons (e.g., false discovery rate method), and biological interpretation can be further strengthened by examining commonalities in clustered populations. Association with survival, if available, can be tested through the Cox proportional hazard methodology, although with many populations special care must be taken to exclude spurious correlations. Machine learning algorithms on larger cohorts may improve the categorical resolution of post‐flow cytometry analysis and feature selection (and can be applied if desired), however, the simple approach described here provides relevant information to formulate testable biological hypotheses at a speed and level of flexibility otherwise attainable only by investigators with substantial computational expertise.

The quality and standardization of the flow cytometry data are crucial to draw valid conclusions from flow cytometry‐based analysis. All of the general rules for standardization of flow cytometry data acquisition apply here: special care should be taken during acquisition, especially in longitudinal studies. Bead‐based standardization is strongly recommended. It is to be noted that the program and subsequent analyses such as PCA and hierarchical clustering—given their unbiased character—can often very efficiently detect systemic biases.

While there theoretically is no limitation to the number of markers that can be computed with *Flow Plex*, the number of input values (obtained through batching) also rises in an exponential fashion, however, on a lower scale. To directly demonstrate the feasibility of the method with standard computational power, we plotted the execution times vs increasing numbers of markers for MarkerCombosBatch (execution time for MarkerCombosInputGeneratorFlowJo is negligible), demonstrating that analyses for up to 10 markers require less than a minute for subpopulation generation, even for large numbers of samples (Figure S11). Clustering methods may become infeasible with a high number of markers without prior feature selection. If manual gating is used, the method probably works best at an optimum in the 4 to 10 marker range (representing 80‐59 048 cell populations per sample), a number of markers often used in clinical studies. By using only a few markers, we could generate meta‐data that helped categorize clinical samples with a high degree of accuracy, even in samples where relatively subtle differences are expected (e.g., young vs old). Also of importance is that many immunologically relevant markers do not follow clear bivariate partitioning, that is, they are expressed as a continuum instead of one positive population distinct from autofluorescence or other background signals. The strict application of FMO (*n*−1) controls helps to clearly differentiate positive from negative signals and is highly recommended when generating data sets to be analyzed with this approach. Considering the former, this method will not discriminate between low/dim vs high/bright vs expression levels of antigens, however, understanding these limitations will enhance its useful application as a discovery tool, stimulating, not replacing, hypothesis‐driven research.

## CONFLICT OF INTERESTS

The authors declare that there are no conflicts of interests.

## Supporting information

Supporting information

Supporting information

Supporting information

Supporting information

Supporting information

Supporting information
