# Supplementary material for: Flow Plex—A tool for unbiased comprehensive flow cytometry data analysis
Source: Immun Inflamm Dis. 2019 Apr 23;7(3):105–11. doi: 10.1002/iid3.246 (PMC6688088; doi:10.1002/iid3.246)

**INSTRUCTIONS FOR RUNNING THE PROGRAM**

**Preliminaries**

Perform analysis of flow cytometry data as described in the Workflow and Supplementary Figure 7. Export the analysis table as an Excel or text file for easy copy/ pasting into the 4markers.csv file below.

**INSTALLING THE PROGRAM**

**Windows**

1. Download the associated files ‘**MarkerCombosInputGeneratorFlowJo.java**’ and ‘**MarkerComboBatch.java**’.

**IMPORTANT:** For countries using numerals with decimal comma (e.g. ‘10,9’ rather than ‘10.9’)( https://en.wikipedia.org/wiki/Decimal_separator), please download ’**MarkerCombosInputGeneratorFlowJo_DecimalComma.java**’ as well as ‘**MarkerComboBatch_DecimalComma.java**’ and use these file names when compiling and executing the program.

Move the file to a desired location if necessary (e.g. a folder containing all future analyses, including input and output data).

1. Identify the path to the directory from which you will run all of your data. This directory should contain both programs **"MarkerCombosInputGeneratorFlowJo.java”** which generates an input file for **"MarkerCombosBatch.java"**, the second program, which computes all possible populations with the markers defined.

To identify the path, right-click on either one of the downloaded files, click ‘Properties’. Click and mouse over the field next to the ‘Location’ section to highlight the selection (or write it down). Right-click and select ‘Copy’. This is the path to the directory containing the files.

For example, for files located on the Desktop, the path may look like ‘C:\Users\yourname\Desktop’

1. Click on the Window ‘start’ icon at the bottom left of the screen, click ‘Run’, type ‘cmd’ and press enter. This opens the command prompt window. This is where the program is installed and will be executed.

Alternatively, press the ‘Windows’ and ‘R’ keys at the same time, and type ‘cmd’.

1. In the command prompt window, type ‘cd’, space, and then paste or type the path to the previously selected directory. This will look like ‘cd C:\Users\yourname\Desktop’.

The program needs to be compiled the first time before being used:

1. Type javac MarkerCombosInputGeneratorFlowJo.java
2. Type javac MarkerCombosBatch.java

NB: for countries using numerals with decimal comma, type:

javac MarkerCombosInputGeneratorFlowJo_DecimalComma.java

and

javac MarkerCombosBatch_DecimalComma.java

**Mac**

1. Download the associated files ‘MarkerComboBatch.java’ and ‘MarkerCombosInputGeneratorFlowJo.java’

Move the file to desired location if necessary (e.g. a folder containing all future analyses, including input and output data).

**IMPORTANT:** For countries using numerals with decimal comma (e.g. ‘10,9’ rather than ‘10.9’)( https://en.wikipedia.org/wiki/Decimal_separator), please download ’**MarkerCombosInputGeneratorFlowJo_DecimalComma.java**’ as well as ‘**MarkerComboBatch_DecimalComma.java**’ and use these file names when compiling and executing the program.

1. Identify the path to the directory in which you will run all of your data. This directory should contain both programs,**"MarkerCombosInputGeneratorFlowJo”** which generates an input file for **"MarkerCombosBatch"**, the second program, which computes all possible populations with the markers defined.

To identify the path, right-click on either one of the downloaded files, click ‘Get info’ (alternatively, click on ‘File’ in the Finder and select ‘Get info’, or simply click ‘Apple’ + ‘I’). Click and mouse over the field next to the ‘Where’ section to highlight the selection (or write it down). Right-click and select ‘Copy’. This is the path to the directory containing the files.

For example, for files located on the Desktop, the path may look like ‘/Users/yourname/Desktop’

1. Open the Terminal. It is located in the *Applications* -> *Utilities* folder.
2. Go into the directory where you will run your data by typing for example:

Type ‘cd’, space, and then paste or type the path to the previously selected directory. This will look like: cd / Users/yourname/Desktop

1. Before using the program for the first time, you will have to compile it by typing:

javac MarkerCombosInputGeneratorFlowJo.java

javac MarkerCombosBatch.java

NB: for countries using numerals with decimal comma, type:

javac MarkerCombosInputGeneratorFlowJo_DecimalComma.java

and

javac MarkerCombosBatch_DecimalComma.java

**RUNNING THE PROGRAM**

1. In the cmd prompt (on Windows) or in Terminal (Mac), make sure that you have specified the working directory containing the ‘MarkerComboBatch.java’ and ‘MarkerCombosInputGeneratorFlowJo.java’ files (use the ‘cd’ command as specified above), as well as the input data.
2. Run the first program, "MarkerCombosInputGeneratorFlowJo", by typing

‘java MarkerCombosInputGeneratorFlowJo M1 M2 M3 M4 > 4markers.csv’

(the names M1 to M4 are just examples, to be replaced by actual marker names)

The input for this program is a list of marker names separated by spaces.

The output is a file name in .csv format. For example, if you use 4 markers:

java MarkerCombosInputGeneratorFlowJo M1 M2 M3 M4 > 4markers.csv

The output is a file named ‘4markers.csv’ that appears in the same working directory.

NB: for countries using numerals with decimal comma, type:

java MarkerCombosInputGeneratorFlowJo_DecimalComma M1 M2 M3 M4 > 4markers.csv

1. Open the file ‘4markers.csv’. Fill in the "FlowValue" columns in the .csv file with the data retrieved from FlowJo, by pasting percentage values from the FlowJo analysis (depending on the table format, you may have to transpose the columns and rows. Save.
2. Run the second program, "MarkerCombos". The input for this program is the .csv file generated by the last program with "FlowValues" filled in. The output is a file name in .csv format. It contains all population values mathematically possible with the number of markers used in the panel.

For example:

java MarkerCombosBatch 4markers.csv > 4markers_output.csv

NB: for countries using numerals with decimal comma, type:

java MarkerCombosBatch_DecimalComma 4markers.csv > 4markers_output.csv

Example:

The AML/ healthy dataset is provided as an example. The proportions of each starting cell subpopulation are provided in the file ‘AML_Healthy_15_13_16_56_All_Pre copy.xlsm’ or ‘AML_Healthy_15_13_16_56_All_Pre_decimal_comma.xlsm’ (for users in countries working with decimal commas). Please download the relevant version of the file and place it in the working directory.

At the cmd prompt or in the Terminal, enter

java MarkerCombosInputGeneratorFlowJo CD13 CD15 CD56 CD16 > 4markers.csv

Open the file ‘4markers.csv’ and paste the percentage values (with disease status) from ‘AML_Healthy_15_13_16_56_All_Pre copy.xlsm’ or – depending on our country-specific settings – ‘AML_Healthy_15_13_16_56_All_Pre_decimal_comma.xlsm’ starting on the 5^th^ column/ 1^st^ row. Save.


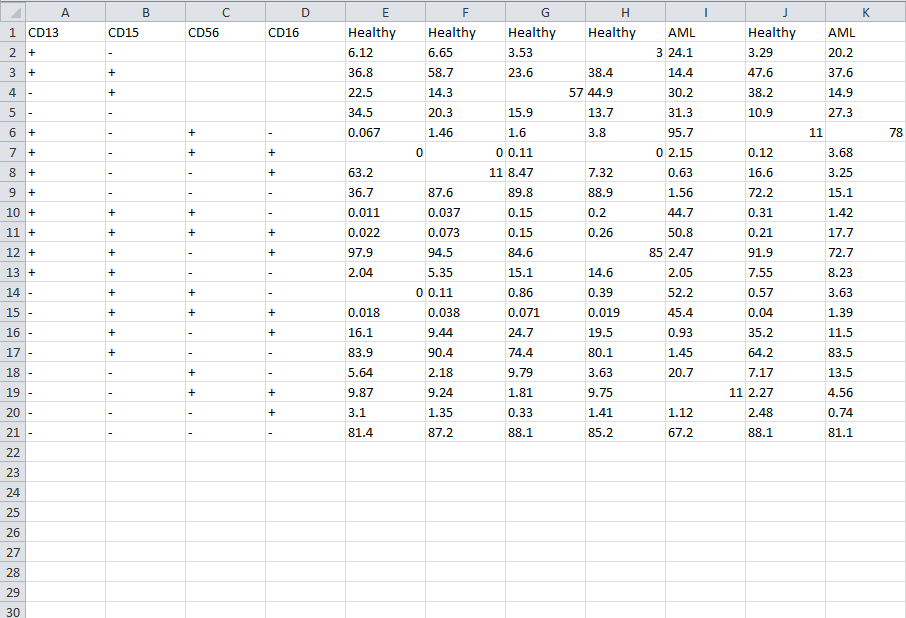


At the cmd prompt or in the Terminal, enter

java MarkerCombosBatch 4markers.csv > 4markers_output.csv

The file ‘4markers_output’ displays the percentages of all subpopulations obtained through combinatorial sub-setting of the different markers (here – given the use of 4 markers – 80 distinct populations per sample). An abbreviated example of the output file format is shown below.


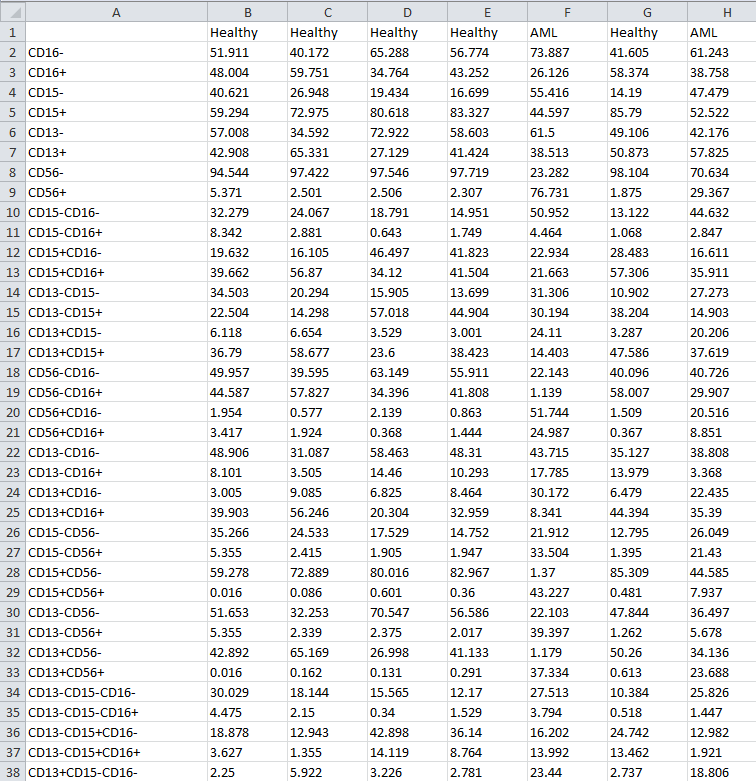

Supplement: Supplementary file 1 — Supporting information [file IID3-7-105-s001.docx]
