# Supplementary material for: Flow Plex—A tool for unbiased comprehensive flow cytometry data analysis
Source: Immun Inflamm Dis. 2019 Apr 23;7(3):105–11. doi: 10.1002/iid3.246 (PMC6688088; doi:10.1002/iid3.246)
Supplement: Supplementary file 6 — Supporting information [file IID3-7-105-s006.pdf]

An unbiased approach to comprehensive flow cytometry data analysis

Johannes Nowatzky[1,*], Esra Reznick[2], Julia Manasson[1], Cristy Stagnar[1], Arshed Fahad Al-Obeidi[1], Olivier Manches[3]

[1]NYU School of Medicine, Department of Medicine, Division of Rheumatology, New York, USA, 10003

[2]Google, Inc., New York, USA, 10011

[3]EFS Rhône-Alpes-Auvergne "Immunobiology and Immunotherapy in Chronic Diseases", INSERM, French National Institute of Health and Medical Research,
Recherche et Développement, “Immunobiology and Immunotherapy in Chronic Diseases”, Institute for Advanced Biosciences, Inserm U 1209, CNRS UMR 5309, Université Grenoble Alpes, Etablissement Français du Sang Auvergne-Rhône-Alpes, 38000 Grenoble-FRANCE Grenoble, France, 38000

[*]Corresponding author: Johannes.Nowatzky@nyumc.org

Supplementary Figure 1

Verification original versus meta-data 4 color panel

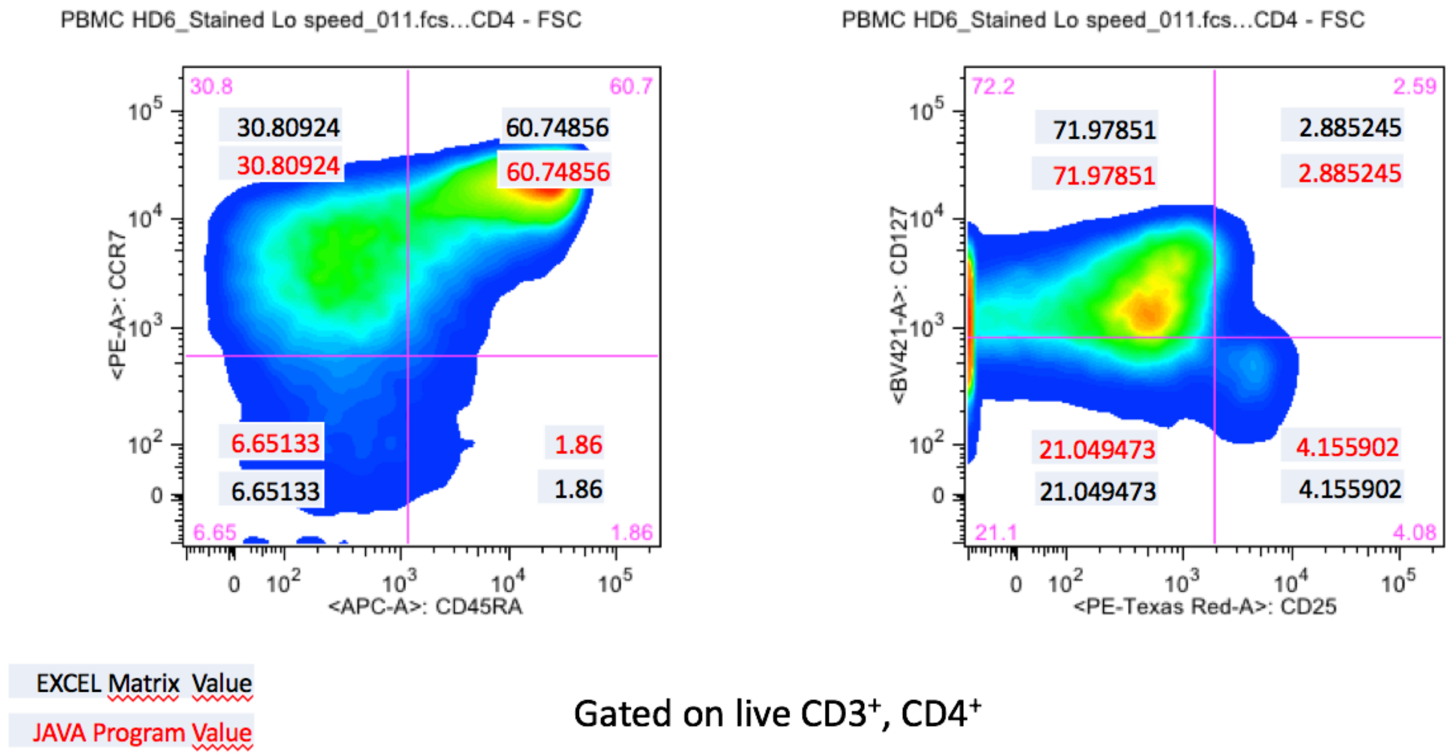

<u>Supplementary Figure 1:</u> Accuracy of computed population frequencies. Four marker analysis (CCR7, CD45RA, CD127, CD25) performed by formula-based spreadsheet (shaded, black print) was compared to direct gating on FlowJo (pink) and 4 marker analysis performed using the Java program (shaded, red print).

Supplementary Figure 2

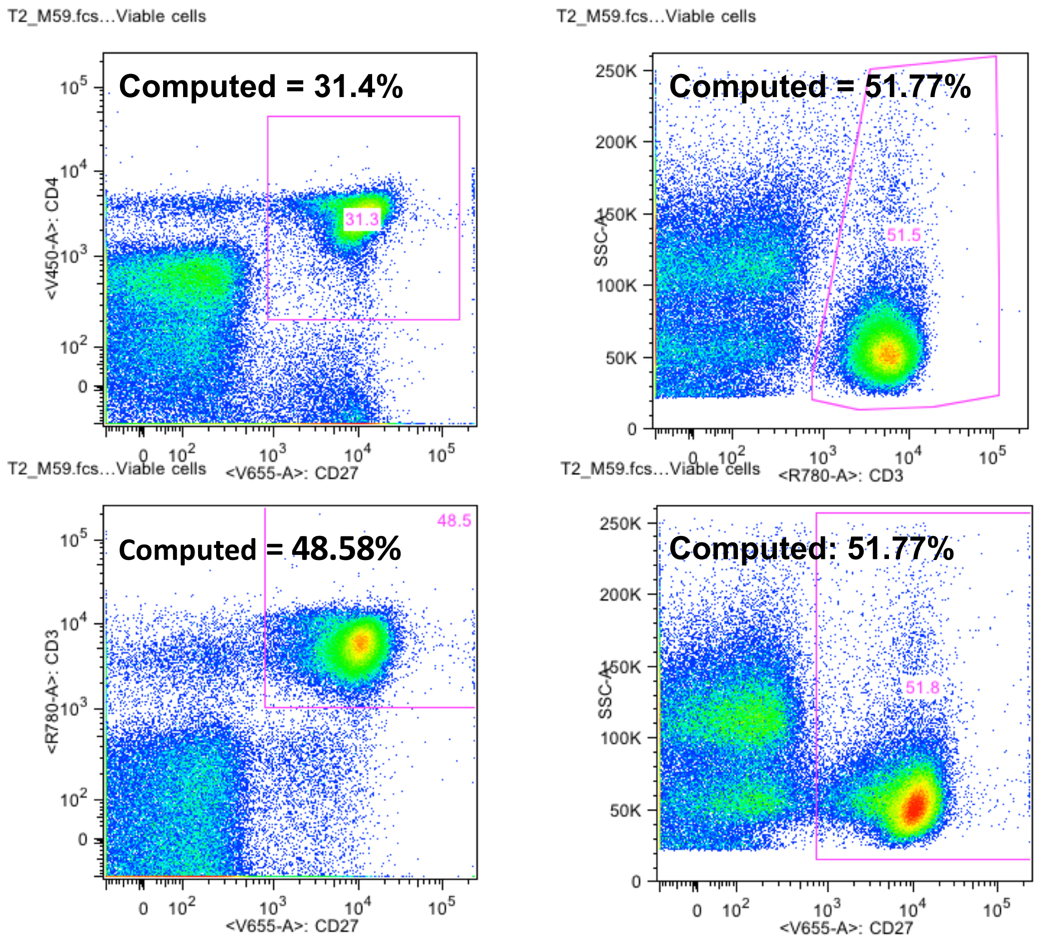

Supplementary Figure 2: Accuracy of computed population frequencies – 6 marker analysis. Six marker analysis was performed using the Java program. Randomly chosen cell populations were gated manually (pink numbers in gates) and contrasted with values computed by the Java program (black print).

Supplementary Figure 3

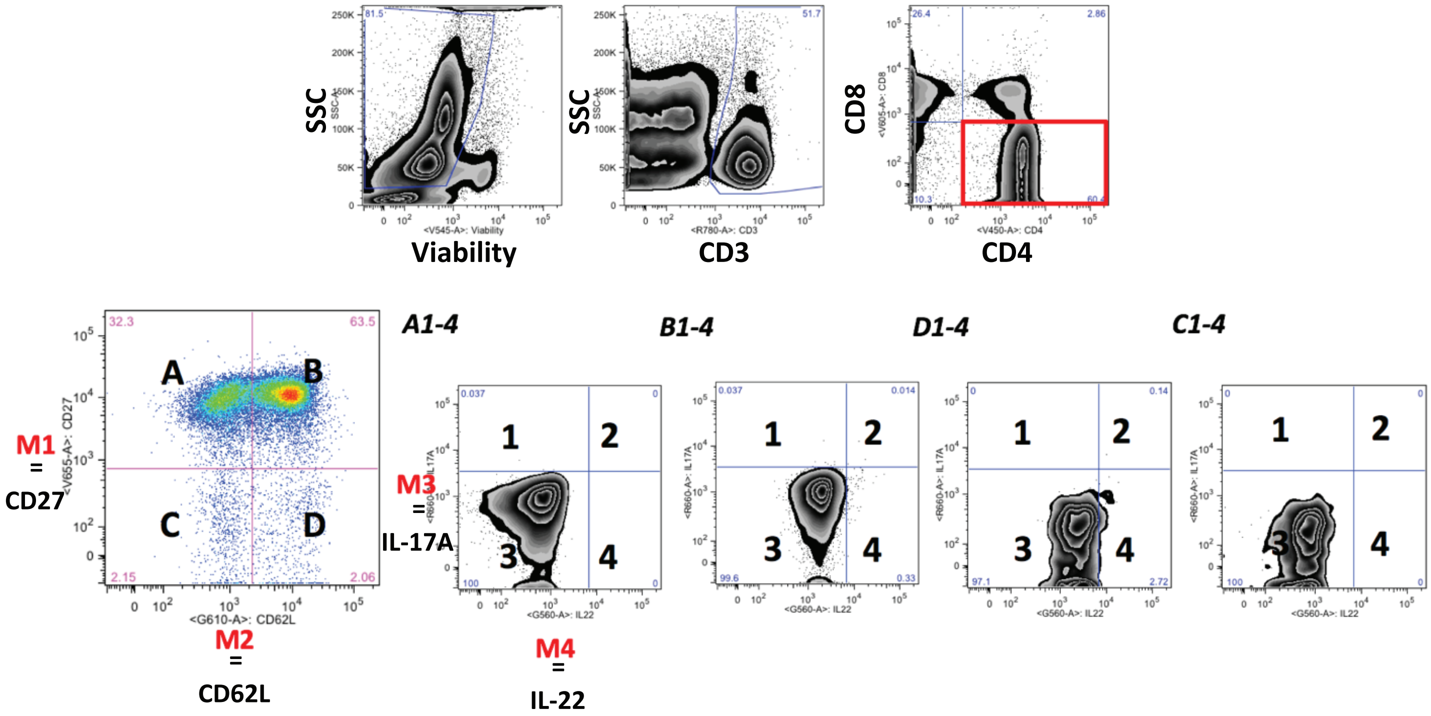

<u>Supplementary Figure 3:</u> Flow cytometry gating strategy for analysis of healthy, BD or SAR samples. Live $CD3^{+}$ $CD4^{+}$ were selected for nested gating using 4 markers (M1 to 4). Quadrant selection for the first two markers (CD27, CD62L) is displayed on the left, generating the A, B, C, D populations. Each subpopulation (e.g. A1 to 4) defined by staining for IL-22 and IL-17A secretion is represented on the right for the A, B, C and D populations.

Supplementary Figure 4

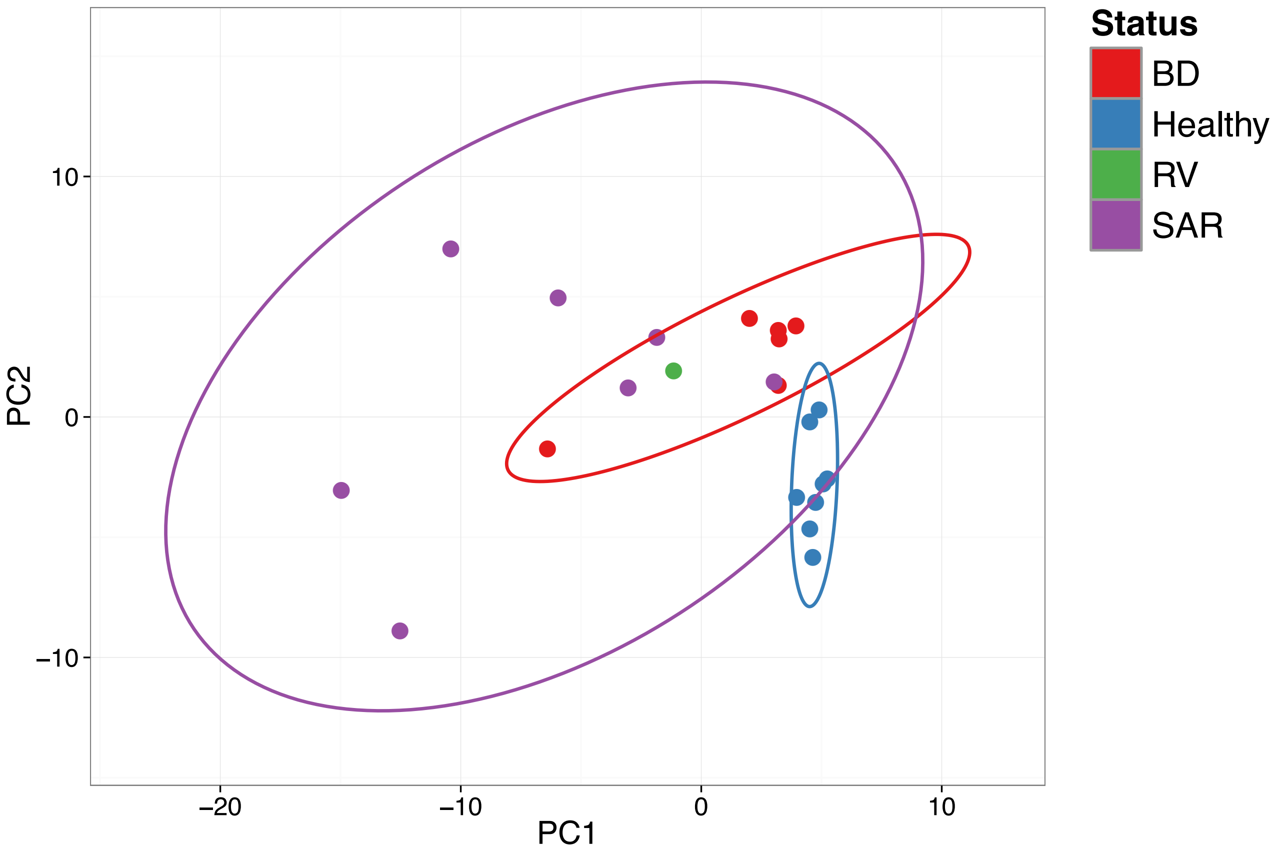

<u>Supplementary Figure 4:</u> Principal component analysis of healthy (blue), Behçet's Disease (BD - red), Sarcoidosis (SAR - purple) or Retinal Vasculitis (RV - green) according to all subpopulations of immune cells in peripheral blood mononuclear cells defined by CD62L or CD27 expression, and IL-22, IL-17A secretion. RV represents an ocular patho-phenotype which is part of the spectrum of both diseases.

Supplementary Figure 5

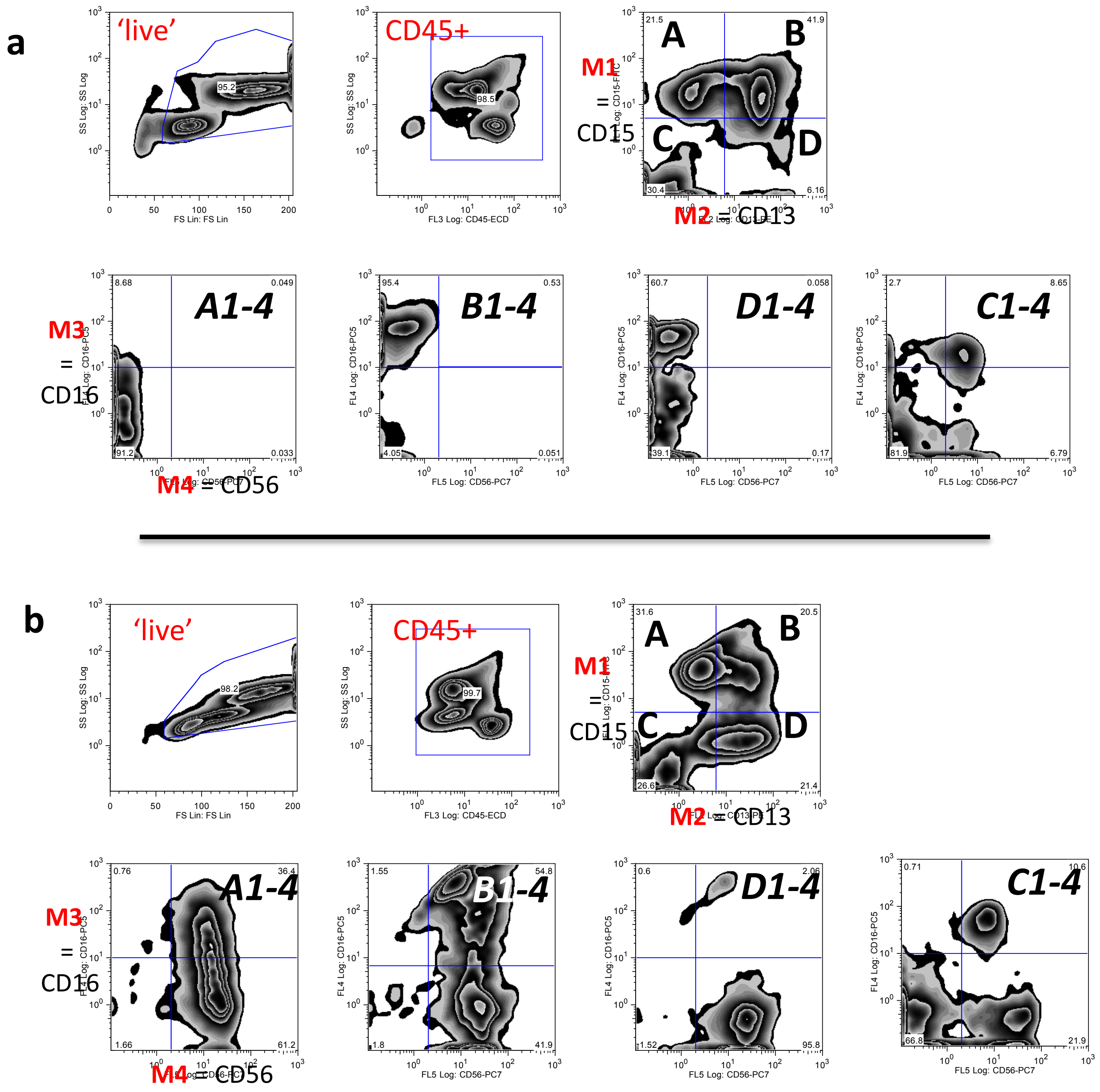

<u>Supplementary Figure 5:</u> Flow cytometry gating strategy for analysis of healthy (a) and acute myeloid leukemia (b) samples. Live CD45^{+} cells were selected for nested gating using 4 markers (M1 to 4). Quadrant selection for the first two markers (CD15, CD13) is displayed on the top right, generating the A, B, C, D populations. Each subpopulation defined by staining for CD16 and CD56 is represented at the bottom for the A, B, C and D populations.

Supplementary Figure 6

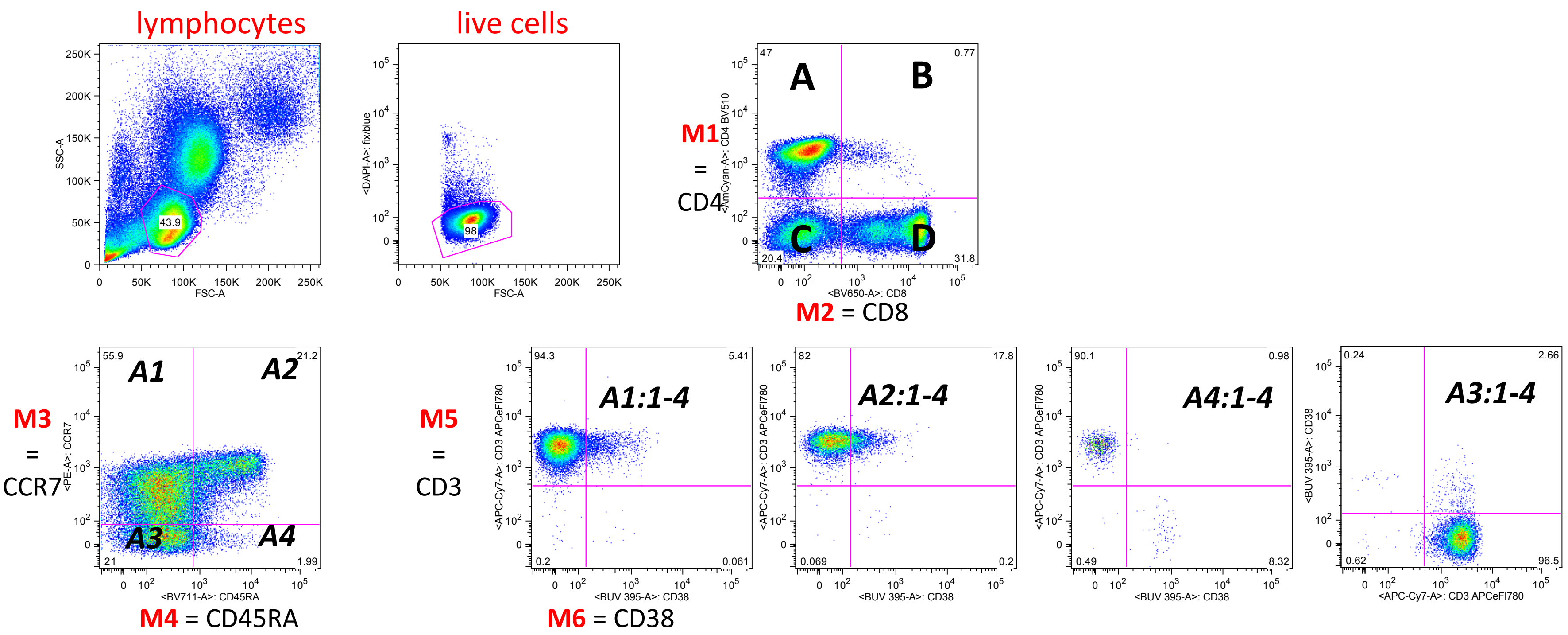

<u>Supplementary Figure 6:</u> Exemplary flow cytometry gating strategy for a 6 marker analysis. Live lymphocytes gated on CD4 versus CD8 are displayed on the top right, generating the A, B, C, D populations. The subpopulation derived from A and defined by staining for CCR7 and CD45RA is selected for nested gating using 6 markers (M1 to 6). Quadrant selection for the first two is shown at the bottom left (A1 to 4). On the bottom right, subpopulations defined by staining with CD3 and CD38 are displayed for A1 to A4 populations. Other subpopulations derived from B, C, and D, are omitted for clarity.

Supplementary Figure 7

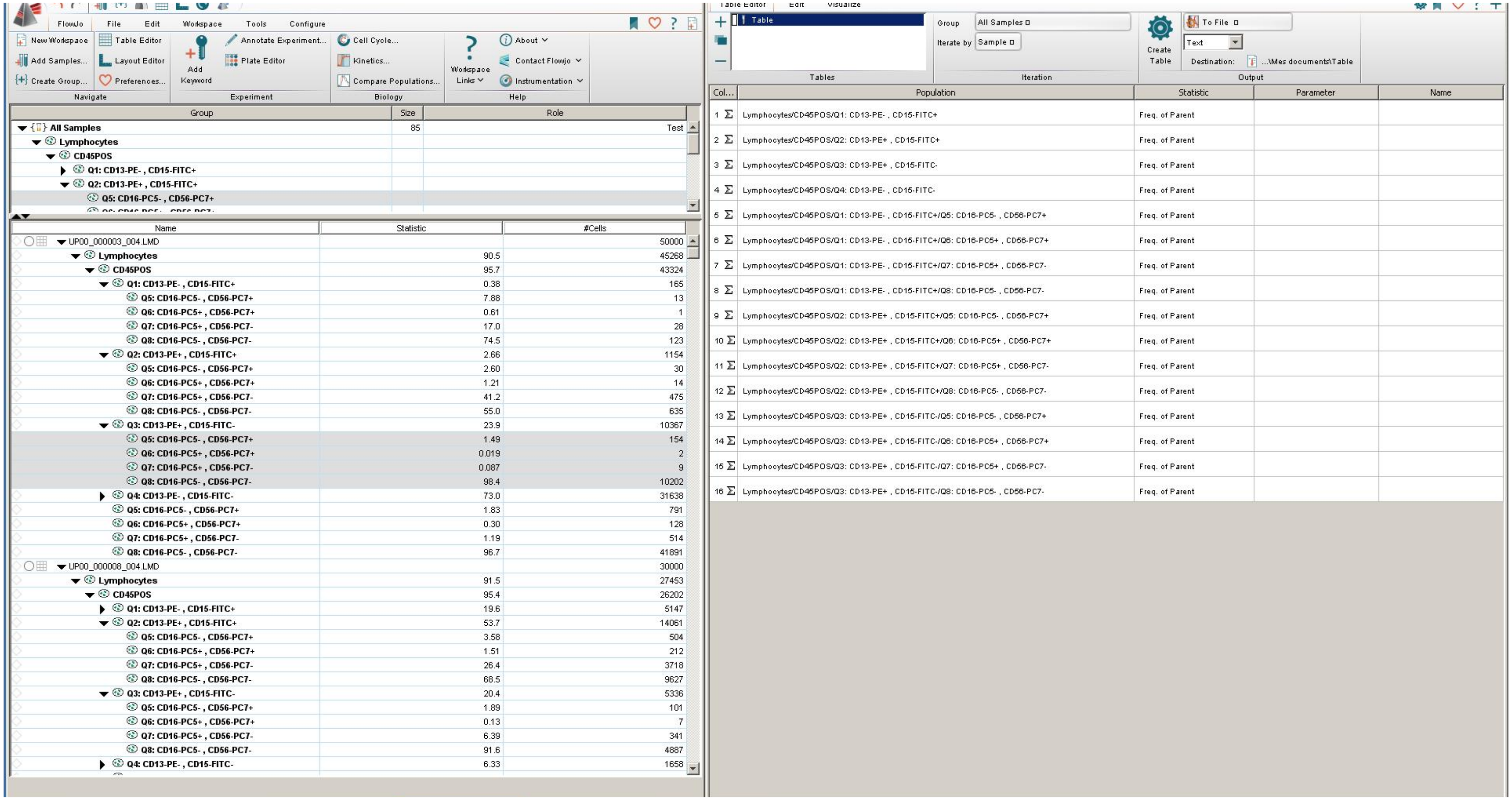

<u>**Supplementary Figure 7:**</u> ***Workflow Step 1***: Gating strategy and table creation for a 4 marker analysis. Quadrants are set to differentiate positive and negative populations for markers 1 and 2, as well as for markers 3 and 4. Quadrants defined for markers 3 and 4 are nested into the quadrants defined by markers 1 and 2. A table is created in a hierarchical way, i.e. Q1 to Q4 first, and Q5 to Q8 within the first quadrants.

Supplementary Figure 8

Input

FSC	SSC	CD15	CD13	CD16	CD56	HD 4	HD 12	HD 20	HD 28	AML 36	HD 44	AML 52
-	-					4.55	30.2	19.3	7.9	7.33	4.54	11.8
-	+					0.91	39.7	20.8	2.25	1.83	1.85	2.12
+	-					3.07	1.73	4.74	1.23	0.63	0.93	1.91
+	+					91.5	28.3	55.1	88.6	90.2	92.7	84.2
-	-	-	-			85.1	98.5	30.9	96.2	96.6	93.6	96
-	-	-	+			1.03	0.062	18.5	1.15	1.02	0.19	0.61
-	-	+	-			12.9	1.44	44.8	2.31	1.32	5.22	2.99
-	-	+	+			1.03	0.031	5.88	0.38	1.08	0.95	0.4
-	+	-	-			82.4	95	11.1	70	85.9	72.1	69.3
-	+	-	+			1.04	0.047	23.6	0.81	2.4	0	6.22
-	+	+	-			16.1	4.9	45.9	27.6	11	25.8	18.7
-	+	+	+			0.52	0.029	19.5	1.62	0.72	2.09	5.82
+	-	-	-			8.55	87	6.64	10.4	17.4	32.6	4.44
+	-	-	+			49.8	1.08	58.4	48.5	43.1	37.2	68.9
+	-	+	-			15.4	9.2	16.2	13.9	8.33	15.8	4.67
+	-	+	+			26.3	2.71	18.8	27.2	31.2	14.4	22
+	+	-	-			1.33	76.7	7.01	1.74	2.54	3	1
+	+	-	+			55.3	1.7	35.2	55.7	66.1	57.3	61.6
+	+	+	-			2.76	16.4	23.4	4.4	2.3	3.78	2.4
+	+	+	+			40.6	5.24	34.3	38.1	29.1	36	35
-	-	-	-	-	-	80.8	97.2	86.4	86.8	76.6	68.2	78.2
-	-	-	-	-	+	1.33	0.00785	0.45	0.4	4.21	3.65	0.94
-	-	-	-	+	-	7.52	2.14	3.82	6.97	6.19	17.6	5.55
-	-	-	-	+	+	10.3	0.67	9.29	5.84	13	10.5	15.3
-	-	-	+	-	-	60	100	77.6	100	64.7	100	52.9
-	-	-	+	-	+	30	0	19.1	0	17.6	0	35.3
-	-	-	+	+	-	10	0	2.76	0	17.6	0	5.88
-	-	-	+	+	+	0	0	0.53	0	0	0	5.88
-	-	+	-	-	-	93.6	98.4	98.1	83.3	100	96.4	94
-	-	+	-	-	+	4	0.53	1.14	13.3	0	0	2.41
-	-	+	-	+	-	2.4	1.07	0.79	3.33	0	3.64	1.2
-	-	+	-	+	+	0	0	0	0	0	0	2.41
-	-	+	+	-	-	20	75	39.5	0	11.1	20	45.5
-	-	+	+	-	+	70	25	58.5	100	11.1	70	45.5
-	-	+	+	+	-	10	0	0.67	0	50	0	9.09

Output

	HD 4	HD 12	HD 20	HD 28	AML 36	HD 44	AML 52
SSC-	7.62	31.94	24.06	9.13	7.96	5.47	13.71
SSC+	92.39	68.01	75.87	90.79	92.10	94.59	86.33
FSC-	5.46	69.92	40.13	10.16	9.16	6.39	13.92
FSC+	94.55	30.04	59.79	89.77	90.89	93.67	86.12
CD16-	98.85	98.58	95.99	98.30	96.57	98.41	96.73
CD16+	1.16	1.38	3.94	1.63	3.49	1.66	3.31
CD15-	58.29	91.23	43.08	60.90	71.08	62.11	67.13
CD15+	41.72	8.73	56.85	39.03	28.98	37.95	32.91
CD13-	9.83	97.87	44.31	15.72	13.48	13.03	16.58
CD13+	90.18	2.09	55.61	84.21	86.58	87.03	83.46
CD56-	46.92	98.68	85.11	63.39	66.01	66.47	60.89
CD56+	53.09	1.28	14.82	36.54	34.05	33.59	39.15
				Missing rows			
SSC-CD56-	5.78	31.69	20.93	8.25	6.53	4.61	11.10
SSC-CD56+	1.84	0.26	3.13	0.88	1.43	0.86	2.61
SSC+CD56-	41.14	66.99	64.18	55.14	59.48	61.86	49.80
SSC+CD56+	51.25	1.02	11.69	35.65	32.62	32.74	36.54
SSC-CD13-	5.19	31.85	15.69	8.08	7.34	4.93	11.85
SSC-CD13+	2.43	0.09	8.37	1.05	0.62	0.53	1.86
				Missing rows			
FSC+SSC+CD13+	87.74	1.96	38.29	83.10	85.90	86.46	81.35
FSC-CD15-CD56-	4.20	67.31	14.54	8.79	7.51	4.97	11.05
FSC-CD15-CD56+	0.47	0.20	2.21	0.50	1.26	0.62	1.95
FSC-CD15+CD56-	0.73	2.39	20.11	0.80	0.35	0.76	0.87
FSC-CD15+CD56+	0.06	0.01	3.28	0.07	0.04	0.04	0.05
FSC+CD15-CD56-	30.35	23.64	24.03	45.01	48.33	43.90	40.21
				Missing rows			
FSC+SSC+CD15+CD13-CD16-CD56+	0.20	0.01	0.79	0.89	0.04	0.67	0.26
FSC+SSC+CD15+CD13-CD16+CD56-	0.04	0.04	0.47	0.12	0.22	0.05	0.05
FSC+SSC+CD15+CD13-CD16+CD56+	0.00	0.00	0.02	0.01	0.00	0.00	0.00
FSC+SSC+CD15+CD13+CD16-CD56-	8.69	0.52	12.70	5.54	7.40	13.78	6.81
FSC+SSC+CD15+CD13+CD16-CD56+	28.31	0.92	5.41	28.12	18.40	19.49	22.28
FSC+SSC+CD15+CD13+CD16+CD56-	0.06	0.03	0.56	0.05	0.25	0.05	0.08
FSC+SSC+CD15+CD13+CD16+CD56+	0.08	0.01	0.23	0.05	0.21	0.05	0.30

Supplementary Figure 8: ***Workflow Step 2:*** The input table, containing populations derived from flow cytometry analysis (first rows are displayed), is fed to the Java algorithm to generate values of all populations in each sample. An example of a 6 marker analysis with 728 populations generated is shown. Rows were skipped for presentation.

Supplementary Figure 9

	A	B	C	D	E	F
1	n Marker M	n maker combinations if 4 markers	Population names	Population codes		
2	1	2 x 2*1 = 8				
3		M2 = (x-axis)	M2 pos	B2+B4+B1+B3+D2+D4+D1+D3	51.918207	0.519343196
4			M2 neg	A2+A4+A1+A3+C2+C4+C1+C3	48.050768	0.480656804
5					99.968975	1
6		M1 = (y-axis)	M1 pos	B2+B4+B1+B3+A2+A4+A1+A3	16.059769	0.160647531
7			M1 neg	D2+D4+D1+D3+C2+C4+C1+C3	83.909206	0.839352469
8					99.968975	1
9		M4 = (x-axis)	M4 pos	B2+B4+D2+D4+A2+A4+C2+C4	46.053374	0.460676665
10			M4 neg	B1+B3+D1+D3+A1+A3+C1+C3	53.915601	0.539323335
11					99.968975	1
12		M3 = (y-axis)	M3 pos	B2+B1+D2+D1+A2+A1+C2+C1	95.12484	0.951543616
13			M3 neg	B4+B3+D4+D3+A4+A3+C4+C3	4.844135	0.048456384
14					99.968975	1
15						
16	2	6 x 2*2=24				
17		M2 and M1	M2 pos M1 pos	B2+B4+B1+B3	15.299541	0.153042892
18			M2 pos M1 neg	D2+D4+D1+D3	36.618666	0.366300305
19			M2 neg M1 pos	A2+A4+A1+A3	0.760228	0.007604639
20			M2 neg M1 neg	C2+C4+C1+C3	47.29054	0.473052164
21					99.968975	1
22		M2 and M4	M2 pos M4 pos	B2+B4+D2+D4	9.65145	0.096544453
23			M2 pos M4 neg	B1+B3+D1+D3	42.266757	0.422798743
24			M2 neg M4 pos	A2+A4+C2+C4	36.401924	0.364132212
25			M2 neg M4 neg	A1+A3+C1+C3	11.648844	0.116524592
26					99.968975	1
27		M2 and M3	M2 pos M3 pos	B2+B1+D2+D1	51.7749	0.517909681
28			M2 pos M3 neg	B4+B3+D4+D3	0.143307	0.001433515
29			M2 neg M3 pos	A2+A1+C2+C1	43.34994	0.433633935
30			M2 neg M3 neg	A4+A3+C4+C3	4.700828	0.047022869
31					99.968975	1
32		M1 and M4	M1 pos M4 pos	B2+B4+A2+A4	4.736094	0.047375638
33			M1 pos M4 neg	B1+B3+A1+A3	11.323675	0.113271893
34			M1 neg M4 pos	D2+D4+C2+C4	41.31728	0.413301027
35			M1 neg M4 neg	D1+D3+C1+C3	42.591926	0.426051442
36					99.968975	1
37		M1 and M3	M1 pos M3 pos	B2+B1+A2+A1	16.01734	0.160223109
38			M1 pos M3 neg	B4+B3+A4+A3	0.042429	0.000424422
39			M1 neg M3 pos	D2+D1+C2+C1	79.1075	0.791320507
40			M1 neg M3 neg	D4+D3+C4+C3	4.801706	0.048031962
41					99.968975	1
42		M4 and M3	M4 pos M3 pos	B2+D2+A2+C2	44.60202	0.446158621
43			M4 pos M3 neg	B4+D4+A4+C4	1.451354	0.014518044
44			M4 neg M3 pos	B1+D1+A1+C1	50.52282	0.505384996
45			M4 neg M3 neg	B3+D3+A3+C3	3.392781	0.033938339
46					99.968975	1

	A	B	C	D	E	F
47	3	4 x 2*3 = 32				
48		M2 and M1 and M4	M2 pos M1 pos M4 pos	B2+B4	4.39569	0.043970542
49			M2 pos M1 pos M4 neg	B1+B3	10.903851	0.10907235
50			M2 pos M1 neg M4 pos	D2+D4	5.25576	0.052573911
51			M2 pos M1 neg M4 neg	D1+D3	31.362906	0.313726394
52			M2 neg M1 pos M4 pos	A2+A4	0.340404	0.003405096
53			M2 neg M1 pos M4 neg	A1+A3	0.419824	0.004199543
54			M2 neg M1 neg M4 pos	C2+C4	36.06152	0.360727116
55			M2 neg M1 neg M4 neg	C1+C3	11.22902	0.112325049
56					99.968975	1
57		M2 and M1 and M3	M2 pos M1 pos M3 pos	B2+B1	15.2847	0.152894435
58			M2 pos M1 pos M3 neg	B4+B3	0.014841	0.000148456
59			M2 pos M1 neg M3 pos	D2+D1	36.4902	0.365015246
60			M2 pos M1 neg M3 neg	D4+D3	0.128466	0.001285059
61			M2 neg M1 pos M3 pos	A2+A1	0.73264	0.007328674
62			M2 neg M1 pos M3 neg	A4+A3	0.027588	0.000275966
63			M2 neg M1 neg M3 pos	C2+C1	42.6173	0.426305261
64			M2 neg M1 neg M3 neg	C4+C3	4.67324	0.046746903
65					99.968975	1
66		M2 and M4 and M3	M2 pos M4 pos M3 pos	B2+D2	9.5517	0.095546643
67			M2 pos M4 pos M3 neg	B4+D4	0.09975	0.00099781
68			M2 pos M4 neg M3 pos	B1+D1	42.2232	0.422363038
69			M2 pos M4 neg M3 neg	B3+D3	0.043557	0.000435705
70			M2 neg M4 pos M3 pos	A2+C2	35.05032	0.350611977
71			M2 neg M4 pos M3 neg	A4+C4	1.351604	0.013520235
72			M2 neg M4 neg M3 pos	A1+C1	8.29962	0.083021958
73			M2 neg M4 neg M3 neg	A3+C3	3.349224	0.033502634
74					99.968975	1
75		M1 and M4 and M3	M1 pos M4 pos M3 pos	B2+A2	4.72322	0.047246858
76			M1 pos M4 pos M3 neg	B4+A4	0.012874	0.00012878
77			M1 pos M4 neg M3 pos	B1+A1	11.29412	0.112976251
78			M1 pos M4 neg M3 neg	B3+A3	0.029555	0.000295642
79			M1 neg M4 pos M3 pos	D2+C2	39.8788	0.398911762
80			M1 neg M4 pos M3 neg	D4+C4	1.43848	0.014389264
81			M1 neg M4 neg M3 pos	D1+C1	39.2287	0.392408745
82			M1 neg M4 neg M3 neg	D3+C3	3.363226	0.033642698
83					99.968975	1
84	4	1 x 2*4 =16				
85		M2 and M1 and M4 and M3	M2 pos M1 pos M4 pos M3 pos	B2	4.3911	0.043924628
86			M2 pos M1 pos M4 pos M3 neg	B4	0.00459	4.59142E-05
87			M2 pos M1 pos M4 neg M3 pos	B1	10.8936	0.108969808
88			M2 pos M1 pos M4 neg M3 neg	B3	0.010251	0.000102542
89			M2 pos M1 neg M4 pos M3 pos	D2	5.1606	0.025811008
90			M2 pos M1 neg M4 pos M3 neg	D4	0.09516	0.000951895
91			M2 pos M1 neg M4 neg M3 pos	D1	31.3296	0.31339323
92			M2 pos M1 neg M4 neg M3 neg	D3	0.033306	0.000333163
93			M2 neg M1 pos M4 pos M3 pos	A2	0.33212	0.003322231
94			M2 neg M1 pos M4 pos M3 neg	A4	0.008284	8.28657E-05
95			M2 neg M1 pos M4 neg M3 pos	A1	0.40052	0.004006443
96			M2 neg M1 pos M4 neg M3 neg	A3	0.019304	0.0001931
97			M2 neg M1 neg M4 pos M3 pos	C2	34.7182	0.347289747
98			M2 neg M1 neg M4 pos M3 neg	C4	1.34332	0.013437369
99			M2 neg M1 neg M4 neg M3 pos	C1	7.8991	0.079015515
100			M2 neg M1 neg M4 neg M3 neg	C3	3.32992	0.033309534
101					99.968975	0.974188992

Supplementary Figure 9: Example of manual four-marker analysis. For a few markers, computations are still manageable using formula-based spreadsheets. Displayed here are calculations of population values with 4 markers. Beyond 4 markers the exponential increase in possible combinations requires computation.

Supplementary Figure 10

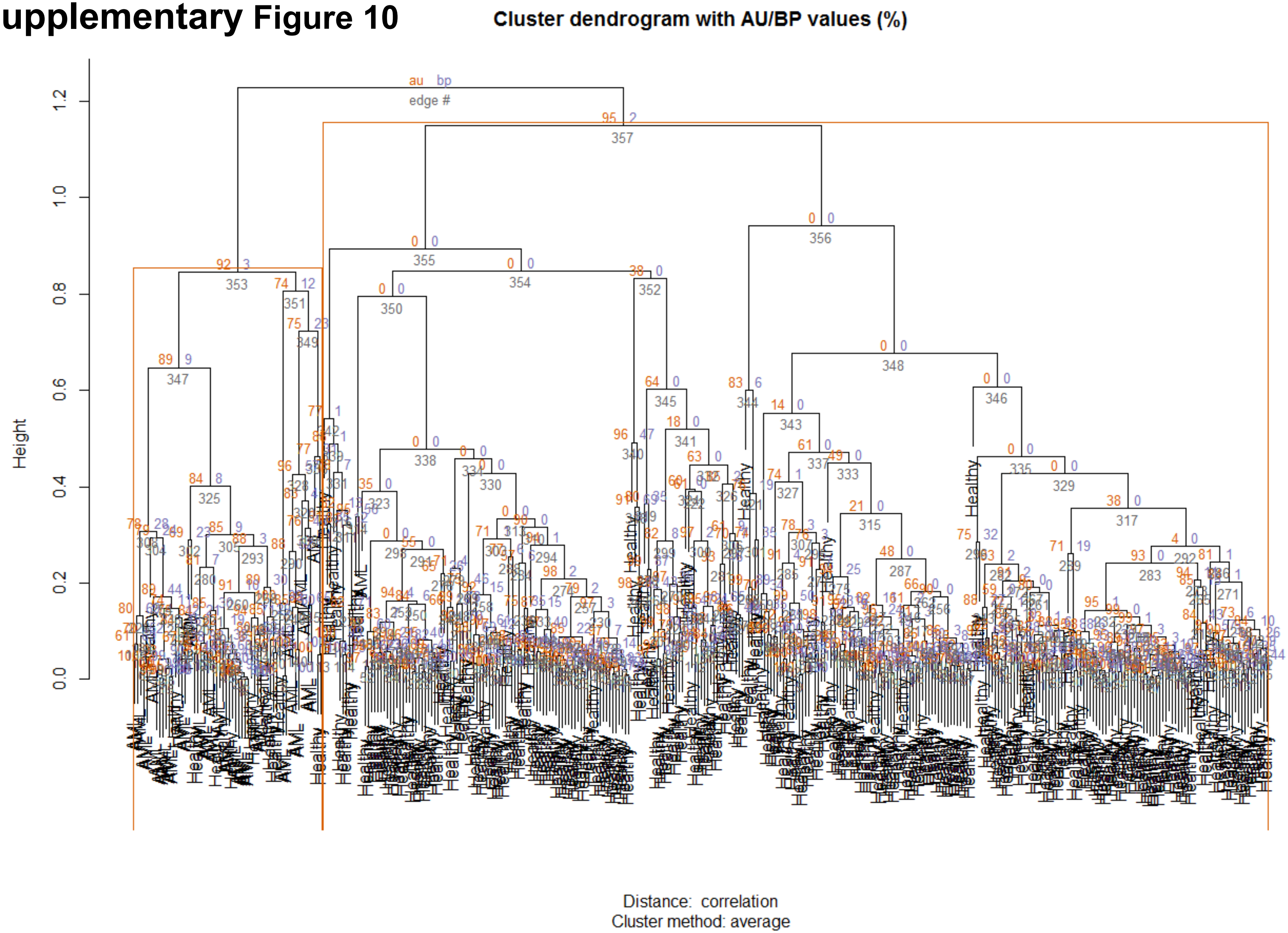

Supplementary Figure 10: ***Statistical analysis of clustering***: The R package pvclust was used to measure the uncertainty of hierarchical clustering for the AML/ healthy dataset based on the 80 populations defined by CD13, CD15, CD16 and CD56 staining. The two main clusters (AML/ healthy) are highlighted in orange. After bootstrapping, AU (Approximately Unbiased) p-values for each cluster is indicated in orange. BP value computed by normal bootstrap resampling are indicated in blue.

Supplementary Figure 11

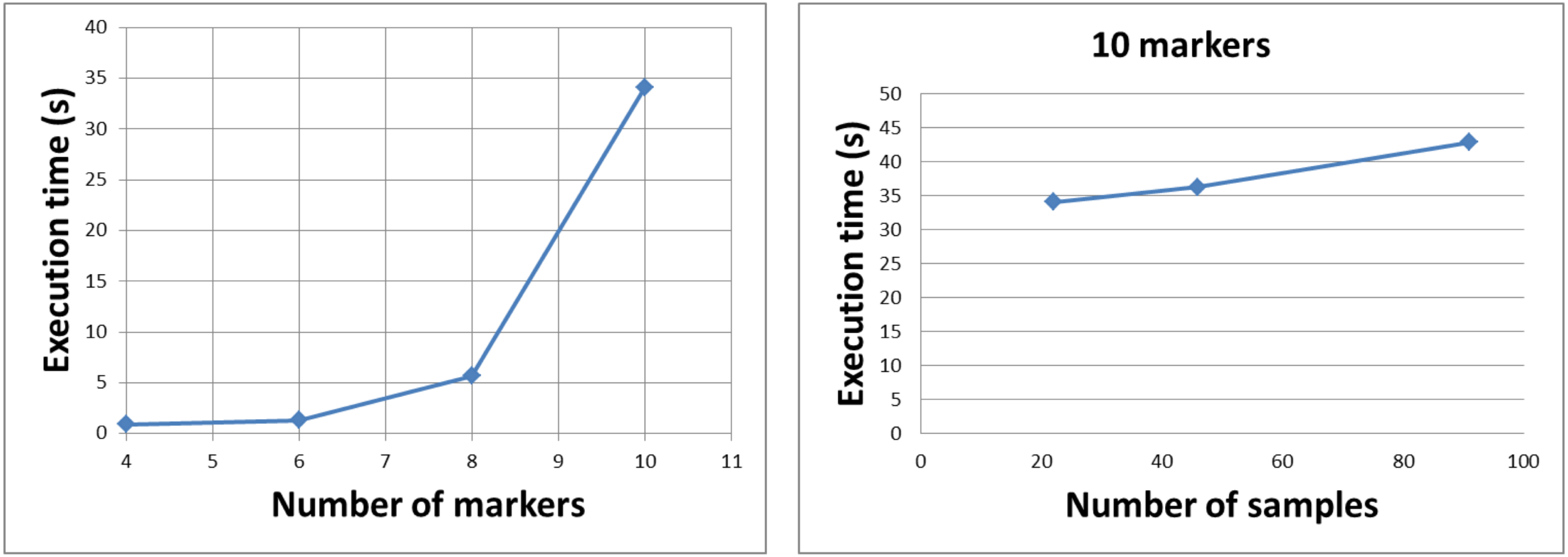

Supplementary Figure 11: ***Execution time with increasing number of markers for MarkerCombosBatch:*** (Left) Execution time for increasing number of 4, 6, 8, and 10 markers for 22 samples, in seconds. Execution time does not exceed 35 seconds. (Right) Execution time for 10 markers and increasing numbers of samples (maximum n=91). Adding more samples to the input file only marginally increases execution time. Calculations were performed on an Intel Pentium Dual CPU E2200 2.20 GHz with 4GB RAM.

Supplementary Table 1

Reagents used for flow cytometry

Item description	Company	Calalogue No	Concentration	Isotype	Clone	Lot. No	Optimal Staining / dilution
PANEL A							
Surface							
**Brilliant Violet 510™** anti-human **CD4** Antibody	Biolegend	344633	25 ug/ml	Mouse IgG1k	SK3	B202014	1 ug/ml
Anti-Human **CD3 APC-eFluor® 780**	eBioscience	47-0038-41	100ug/mL	Mouse IgG1 K	UCHT1	E08436	2 ug/ml
*Brilliant Violet **650™** anti-human **CD8** Antibody*	*Biolegend*	*344729*	*100ug/mL*	*Mouse IgG1, κ*	*SK1*	*B195359*	4 ug/ml
**PE/Dazzle™ 594** anti-human **CD25** Antibody	Biolegend	356125	100 ug/ml	Mouse IgG1, κ	M-A251	B203122	1 ug/ml
**Brilliant Violet 421™** anti-human **CD127 (IL-7Rα)** Antibody	Biolegend	351309	100ug/mL	Mouse IgG1, κ	A019D5	B205136	2 ug/ml
**Brilliant Violet 711™** anti-human **CD45RA** Antibody	Biolegend	304137	25ug/mL	Mouse IgG2b, κ	HI100	B206332	4 ug/ml
**PE** anti-human **CD197 (CCR7)** Antibody	Biolegend	353204	160ug/mL	Mouse IgG2a, κ	G043H7	B197942	2 ug/ml
**BUV395** Mouse Anti-Human **CD38**	BD Biosciences	563812	300 ug/ml	Mouse IgG1k	HB7	602184	1 ug/ml
Viability dye							
Viability dye: **Fixable Blue** (use at 1:1000)	Invitrogen						1:1000

Supplementary Table 2

Donor information

Donor ID	SPECIMEN OBTAINED				
	(Date the blood was obtained from donor)	SEX Male	Female	AGE Range	ACTUAL CONFIRMED AGE AND GENDER
**HDC-8**	2017-1-23	1	0	65-95	M 67
**HDC-9**	2017-1-23	1	0	65-95	M 68
**HDC-10**	2017-1-24	1	0	65-95	M 68
**HDC-11**	2017-1-24	1	0	65-95	M 66
**HDC-12**	2017-1-25	1	0	65-95	M 65
**HDC-13**	2017-1-25	1	0	65-95	M 66
**HDC-14**	2017-1-26	1	0	65-95	M 69
**HDC-15**	2017-1-26	1	0	65-95	M 67
**HDC-29**	2017-4-19	1	0	18-35	M 26
**HDC-30**	2017-4-19	1	0	18-35	M 23
**HDC-31**	2017-4-20	1	0	18-35	M 18
**HDC-32**	2017-4-20	1	0	18-35	M 18
**HDC-41**	2017-6-14	0	1	65-95	F 66
**HDC-42**	2017-6-14	0	1	65-95	F 78
**HDC-43**	2017-6-20	0	1	65-95	F 76
**HDC-44**	2017-6-20	0	1	65-95	F 69
**HDC-45**	2017-6-21	0	1	65-95	F 65
**HDC-46**	2017-6-21	0	1	65-95	F 74
**HDC-47**	2017-6-28	0	1	65-96	F 75
**HDC-48**	2017-6-28	0	1	65-97	F 67
**HDC-50**	2017-7-06	0	1	18-35	F 21
**HDC-51**	2017-7-06	0	1	18-35	F 23
**HDC-53**	2017-7-13	0	1	18-35	F26
